# Under the Surface: A Case of Secondary Delusional Infestation With Mood-Congruent Psychotic Features in a 56-Year-Old Woman

**DOI:** 10.7759/cureus.112268

**Published:** 2026-07-08

**Authors:** Angela Chen, Travis Brauer

**Affiliations:** 1 Osteopathic Medicine, Nova Southeastern University Dr. Kiran C. Patel College of Osteopathic Medicine, Clearwater, USA; 2 Psychiatry, Lakeland Regional Health, Lakeland, USA

**Keywords:** acute mania, bipolar i disorder with psychotic features, delusional infestation, formication, history of bipolar disorders, skin excoriation, stimulant-induced psychosis

## Abstract

Delusional infestation (DI) is an uncommon phenomenological syndrome characterized by the fixed, false conviction of being infested by living organisms, which often results in significant disease burden through social isolation and self-destructive behaviors. This case explores a 56-year-old woman with HIV and a history of bipolar disorder who presented with suicidal ideation and profound distress attributed to a believed parasitic infestation following a recent dose escalation of her prescribed amphetamine. The patient exhibited classic manic features, including hypergraphia and pressured speech, alongside formication and extensive scalp excoriations complicated by a secondary *Pseudomonas aeruginosa* wound infection. A thorough workup, including a CD4 count of 515 cells/μL and neuroimaging, effectively excluded organic etiologies and HIV-associated opportunistic infections, confirming a diagnosis of bipolar I disorder, current manic episode with mood-congruent psychotic features, with delusional infestation as the predominant psychotic content, likely exacerbated by stimulant uptitration. Management focused on a multidisciplinary approach utilizing divalproex sodium for mood stabilization and olanzapine for its antipsychotic and sedative effects, while amphetamines were discontinued. By employing a communication strategy of “empathetic neutrality,” which involved validating the patient’s distress without confirming the delusion, the clinical team established a therapeutic alliance that fostered cognitive flexibility. By discharge, the patient demonstrated a significant reduction in distress and improved functionality, even as a degree of delusional conviction persisted. This case highlights the importance of the dopaminergic hypothesis in DI pathophysiology and illustrates that clinical success in such complex cases is often defined by reduced morbidity and improved quality of life rather than the total eradication of the delusion. It contributes to the limited literature regarding the interplay of comorbid bipolar disorder, stimulant use, and secondary DI.

## Introduction

Delusional infestation (DI), historically known as Ekbom syndrome, is a rare phenomenological syndrome characterized by the fixed, false belief that one is infested by living organisms, such as parasites, worms, mites, bacteria, or fungi. While the term “Ekbom syndrome” is still used, “delusional infestation” is increasingly preferred in the literature to reflect the diverse range of pathogens reported by patients.

The condition is categorized into two subtypes: primary DI, in which the delusion presents as the sole psychiatric manifestation, and secondary DI, in which the delusion occurs as a symptom of an underlying condition [1]. The latter may arise from other psychiatric disorders, organic medical conditions such as cerebrovascular disease or Parkinson’s disease, or as a direct result of substance use and abuse.

DI carries a significant disease burden, often leading to social isolation and self-destructive behaviors, such as skin excoriation or the application of caustic substances. While some patients may maintain a degree of outward functionality, the condition frequently results in high healthcare utilization and significant distress. Although delusions often resist medical evidence, leading patients to seek help from dermatologists rather than psychiatrists, treatment can significantly reduce distress and improve quality of life, even when the underlying conviction remains [2].

While the condition has been recognized since the 19th century and more than 1,400 cases have been documented, high-quality evidence from controlled trials is lacking [3]. Most existing literature is limited to case reports and observational studies. According to a retrospective study by the Mayo Clinic, the typical patient profile includes a mean age of 57 years, with a female-to-male ratio of 2.9:1 [4].

The exact pathophysiology of DI remains unknown, though two main theories have been proposed. The somatic amplification theory posits that the belief may arise from the amplification of somatic symptoms triggered by illness anxiety and the misinterpretation of normal bodily sensations [4]. A more specific neurobiological theory, the dopamine transporter (DAT) hypothesis, suggests decreased striatal DAT functioning, leading to increased extracellular dopamine levels in the striatum rather than an increase in dopamine release itself [5,6]. This mechanism is particularly relevant in cases involving substances such as amphetamines, which act as DAT inhibitors [5,6]. Neuroimaging support for this hypothesis includes PET studies showing elevated dopamine synthesis capacity in the caudate region of affected patients [7].

A clinical hallmark feature of DI is the “specimen sign,” in which patients bring physical “evidence” of their supposed infestation, typically skin particles, hair, or debris, to clinicians as proof. A European multicenter study of 148 consecutive DI cases found that 48% of patients presented such specimens, and the authors recommended the term “specimen sign” to replace the older “matchbox sign,” which they found no longer reflected common presentation [8].

This report aims to contribute to the understanding of DI by presenting a representative case, with a thorough discussion of the observed clinical course, diagnostic challenges, and therapeutic considerations.

## Case presentation

The patient was a 56-year-old woman with a medical history of HIV and a psychiatric history notable for ADHD and a reported remote history of bipolar disorder. She was a limited retrospective historian, and clinical assessment was further hindered by a lack of corroborating outside records. She was admitted to an inpatient psychiatric facility following an emergency department presentation for suicidal ideation, which she attributed to the profound distress caused by her believed parasitic infestation.

One month prior to admission, the patient’s provider uptitrated her amphetamine/dextroamphetamine dose to 30 mg daily. Subsequently, she began experiencing significant insomnia, averaging two to three hours of sleep per night, and a 10-pound weight loss, yet she maintained high energy levels. During this period, she authored a 100-page manuscript on the works of Carl Jung despite having no formal education in psychology, a finding indicative of hypergraphia and goal-directed hyperactivity.

Her psychosocial history was marked by several acute stressors, including a recent job loss following an ethical dispute at work and the impending third anniversary of her son’s death. Her medical history was significant for HIV; however, her CD4 count of 515 cells/μL effectively excluded HIV-associated opportunistic CNS infections as a primary cause of her psychosis. Her opioid use disorder had been stable on buprenorphine-naloxone for the past year, although she had been transitioned to methadone at a previous facility for reasons that remained unclear. This transition is pharmacologically relevant: methadone carries QTc-prolonging potential, and this risk requires consideration in the context of concurrent antipsychotic initiation.

On admission, the patient was visibly distressed and tearful. She described formication (the tactile hallucination of crawling or biting sensations beneath the skin), claiming that parasites were “crawling” beneath her skin and reporting the removal of “thin red fibers.” She attributed the onset to prior OnabotulinumtoxinA injections and a recent scabies infestation in her late pet. Her delusions were multifaceted: she shaved her head because her hair felt as though it was “moving” and applied iodine to her abdomen to coax “worms” to emerge. She pointed to natural skin contours as “evidence” and, during admission, collected scabs in containers for testing, a presentation consistent with the specimen sign described in the literature [8], stating, “I might be patient zero, but no one is taking me seriously.”

**Figure 1:**
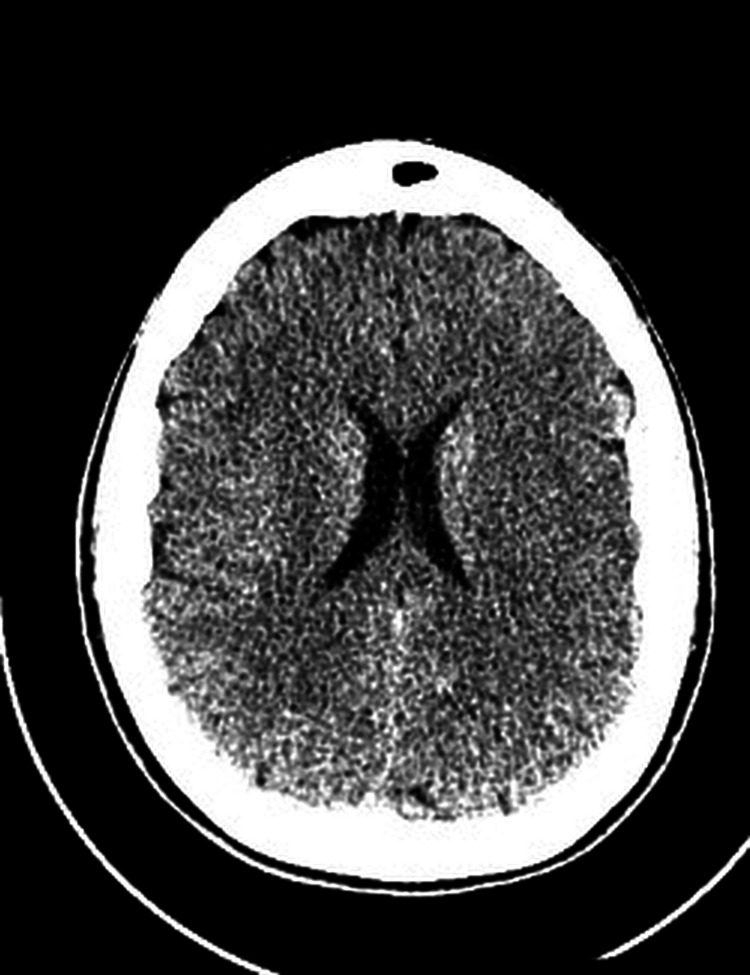
Noncontrast CT of the brain (axial view). The image obtained at the level of the lateral ventricles demonstrates no acute intracranial hemorrhage, mass effect, midline shift, or findings suggestive of HIV-associated opportunistic central nervous system infection. The ventricular system is within normal limits for the patient’s age.

**Figure 2:**
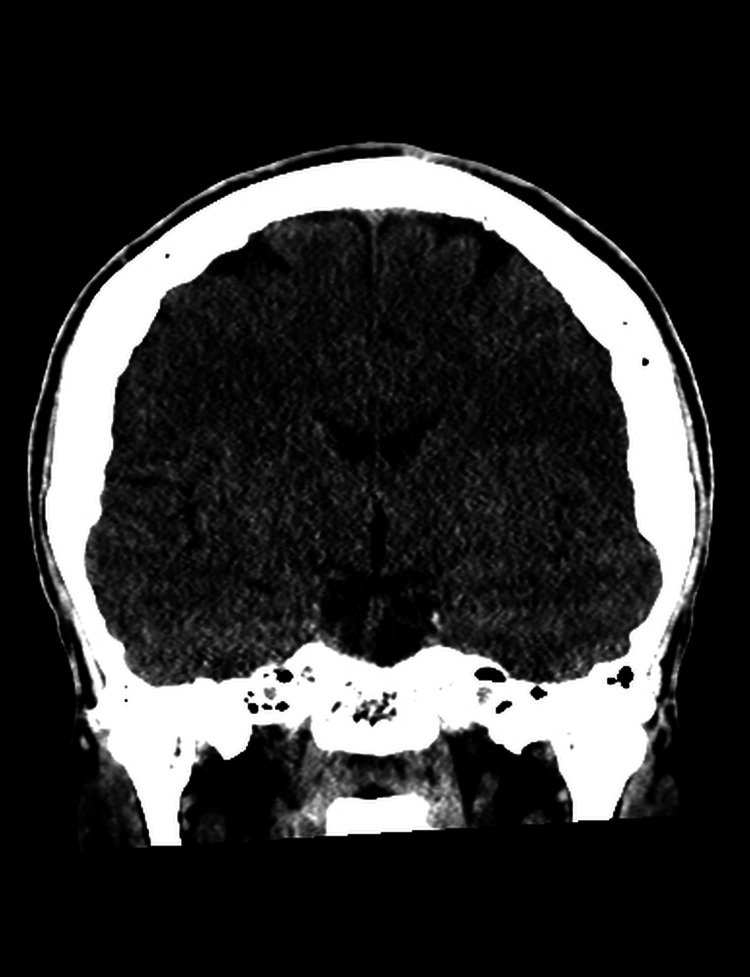
Noncontrast CT of the brain (coronal view). The image demonstrates no acute pathology involving the basal ganglia, thalami, or temporal lobes. No mass lesions, hemorrhage, or focal areas of hypoattenuation are identified. The findings are consistent with the absence of opportunistic infection or other organic etiology accounting for the patient’s acute neuropsychiatric presentation.

Mental status examination revealed pressured speech and linear thought processes with occasional flight of ideas. While she denied active suicidal intent, she acknowledged hopelessness regarding her condition. Physical examination was notable for multiple scalp excoriations in varying stages of healing, consistent with chronic picking. A wound culture obtained from the excoriated scalp grew *Pseudomonas aeruginosa*, representing a secondary bacterial infection complicating the mechanical trauma caused by delusional excoriation behavior. Noncontrast CT of the brain was obtained to exclude organic CNS etiologies and HIV-associated opportunistic infections, demonstrating no acute intracranial pathology, no mass effect, and no findings suggestive of opportunistic infection (Figures 1, 2). Laboratory studies, including syphilis and hepatitis panels, were also unremarkable. A urine drug screen was positive for amphetamines, methadone, and cannabis. The complete laboratory workup and reference ranges are summarized in Table 1.

**Table 1:** Summary of laboratory and diagnostic investigations performed during admission. Routine metabolic and hematologic investigations (complete blood count, basic metabolic panel, thyroid-stimulating hormone, and vitamin B12) were within reference ranges on admission. Exact numerical values are unavailable for retrospective reporting.

Parameter	Patient value	Reference range
CD4 count	515 cells/μL	500-1500 cells/μL
HIV viral load	Within the reference range	<20 copies/mL (undetectable)
Complete blood count (WBC)	Within the reference range	4.5-11.0 × 10⁹/L
Basic metabolic panel	Within the reference range	Within the reference range
Thyroid-stimulating hormone	Within the reference range	0.4-4.0 mIU/L
Vitamin B12	Within the reference range	200-900 pg/mL
Rapid plasma reagin/syphilis	Nonreactive	Nonreactive
Hepatitis B surface antigen	Negative	Negative
Hepatitis C antibody	Negative	Negative
Urine drug screen	Positive for amphetamines, methadone, and cannabis	Negative
Wound culture (scalp)	Pseudomonas aeruginosa	No growth
Noncontrast CT brain	Unremarkable (see Figures 1, 2)	No acute findings

The clinical priority was differentiating between stimulant-induced psychosis and bipolar mania with psychotic features. While the temporal relationship between amphetamine uptitration and symptom onset suggested a stimulant contribution, the patient met full DSM-5-TR criteria for bipolar I disorder, current manic episode, with mood-congruent psychotic features (ICD-10-CM F31.2; DSM-5-TR 296.44) [9]. The required specifiers were present: decreased need for sleep, hypergraphia, pressured speech, and flight of ideas constituted the manic episode, while the fixed somatic delusion of parasitic infestation represented mood-congruent psychotic content. Although Substance/Medication-Induced Bipolar and Related Disorder (DSM-5-TR 292.84) [9] was considered given the temporal proximity to amphetamine uptitration, this diagnosis is most appropriate when the mood disturbance does not persist beyond the direct physiological effects of the substance. Given the patient’s history of bipolar disorder and the persistence of manic features consistent with prior episodes, Bipolar I Disorder remained the primary DSM-5-TR formulation. It must be acknowledged, however, that this diagnostic distinction rests substantially on the patient’s self-report, as she was a limited retrospective historian with no corroborating outside records. The possibility of substance-induced mania cannot be fully excluded, and this diagnostic uncertainty should temper the confidence with which a primary Bipolar I formulation is asserted. Stimulant use was identified as a significant precipitating and exacerbating factor in either case. “Delusional infestation” is used throughout this report as a phenomenological descriptor of the psychotic content, specifically formication and fixed somatic beliefs, rather than as a standalone DSM-5-TR diagnosis.

On hospital day 1, she was started on divalproex sodium ER 500 mg BID for mood stabilization and standard olanzapine 10 mg nightly. Notably, the combination product olanzapine/samidorphan (Lybalvi) is contraindicated in patients using opioids or undergoing opioid withdrawal due to the samidorphan component; selection of standard olanzapine in this patient, who was receiving methadone, appropriately avoided this interaction. On hospital day 2, the patient remained preoccupied with “specimen collecting” (saving scabs for testing). She accepted a protective head covering to prevent further trauma. To address emerging akathisia and residual anxiety, propranolol 30 mg BID was initiated.

By day 3, the patient demonstrated the first signs of cognitive flexibility. Following a strategy of empathetic neutrality by the clinical staff, validating her distress without confirming the delusion, she acknowledged for the first time that her symptoms might have a psychiatric etiology. Her olanzapine was titrated to 15 mg nightly to further address persistent delusional conviction and insomnia.

By hospital day 5, the patient was socially engaged and significantly less preoccupied with her skin. When staff reassured her that her skin showed no signs of active infestation, she offered an insight-oriented response: “Maybe the parasites are dying.” This shift highlighted the efficacy of the therapeutic approach in reducing the distress associated with the delusion, even as the underlying conviction remained partially intact.

The patient was discharged on hospital day 6 in stable condition. Her discharge regimen included divalproex sodium ER 500 mg BID, olanzapine 15 mg nightly, and propranolol 30 mg BID. She was instructed to resume Biktarvy and buprenorphine-naloxone but was explicitly advised to discontinue amphetamines, as the stimulant likely precipitated the manic episode. She was discharged to the care of her roommate with referrals for outpatient psychiatric follow-up and psychotherapy.

## Discussion

This case illustrates the diagnostic complexity of differentiating primary from secondary DI in a patient with multiple neuropsychiatric “hits,” including HIV, bipolar mania, and active stimulant use. Her presentation, characterized by fixed beliefs of parasitic infestation, tactile hallucinations, and self-inflicted excoriations, provides a unique look into how overlapping etiologies can manifest as a singular somatic delusion. The multifactorial etiology in this patient underscores the importance of the DI classification framework, in which primary DI exists as a monosymptomatic disorder and secondary DI is driven by identifiable medical conditions, substances, or psychiatric comorbidities [1]. Critically, within a formal DSM-5-TR framework, the patient’s DI is best understood as mood-congruent psychotic content within a Bipolar I manic episode (ICD-10-CM F31.2) rather than a standalone diagnostic entity [9]. Because 42% of DI patients have a comorbid psychiatric condition, treatment must prioritize these underlying causes, such as mood stabilization for bipolar mania, rather than focusing solely on the delusional content [10].

The convergence of bipolar mania, stimulant use, and DI in this patient may plausibly be explained by a shared hyperdopaminergic mechanism, though this remains a hypothesis-generating observation from a single case rather than an established causal pathway. Amphetamines increase synaptic dopamine by blocking the DAT, which, at high levels or during uptitration, can produce formication and the delusional misinterpretation of somatic sensations [11-13]. In this case, stimulant uptitration may have acted as a “double hit,” potentially exacerbating both the mood episode and delusional symptoms through hyperactivation of the mesolimbic pathway [14]. This offers a plausible, though not definitive, rationale for the use of D2 receptor antagonists. While risperidone and olanzapine are among the most commonly described antipsychotics in DI case literature, no randomized controlled trials exist to establish any agent as definitively first-line; evidence consists entirely of case reports and small series [3]. The choice of olanzapine in this case addressed specific clinical needs for sedation and appetite stimulation.

The temporal relationship between the patient’s stimulant uptitration and symptom intensification is a significant clinical finding. A 2025 systematic review and meta-analysis published in JAMA Psychiatry found that 2.8% of individuals with ADHD prescribed stimulants developed psychotic symptoms, with amphetamines associated with 57% higher odds of psychosis compared with methylphenidate [15]. The authors recommended systematic monitoring and consideration of methylphenidate over amphetamines in patients with psychosis risk factors, a recommendation directly applicable here [15]. This is consistent with prior research indicating that 60% of patients with psychocutaneous disorders have recent stimulant use [16]. Although a diagnosis of Substance/Medication-Induced Bipolar and Related Disorder (DSM-5-TR 292.84) was considered, the persistence of manic features and the reported prior bipolar history supported the primary mood disorder diagnosis, with stimulant use as an exacerbating factor. Nonetheless, this case necessitates extreme caution when managing ADHD with stimulants in patients with a history of bipolar disorder. To manage this, the clinical team employed “empathetic neutrality,” focusing on the patient’s emotional distress rather than challenging the logic of the infestation [17]. Framing antipsychotics as a way to address “biochemical imbalances” is a practical strategy that preserves the therapeutic alliance, which is critical given that DI patients characteristically avoid psychiatric labels [17].

DI patients often present first to nonpsychiatric clinicians, frequently bringing “evidence” of infestation, the specimen sign, to dermatologists or infectious disease specialists [8]. Early recognition is vital because 41.5% of these patients are lost to follow-up [18]. While the patient’s mood and distress in this case improved, some delusional conviction persisted, which aligns with literature suggesting that only 24.6% of patients achieve complete remission [18]. Among those who did engage with treatment, patients who took medication and had a planned discharge demonstrated a CGI-S score change of -2.02, indicating clinically significant improvement and reinforcing that reduced morbidity is a realistic and meaningful outcome even when full remission is not achieved [18]. Ultimately, the goal of treatment is the reduction of distress and improvement of quality of life, acknowledging that while the dopaminergic hypothesis is biologically plausible, the lack of randomized controlled trials means management remains heavily reliant on case-based evidence.

## Conclusions

This case highlights the significant diagnostic and therapeutic challenges of secondary DI, a condition frequently first encountered by nonpsychiatric clinicians, including dermatologists, primary care physicians, and infectious disease specialists. The patient’s fixed somatic delusions persisted despite an exhaustive dermatologic, infectious, and neuroimaging workup that yielded no evidence of true infestation. The clinical timeline, characterized by psychosocial stressors and a recent amphetamine uptitration, suggests a presentation of bipolar I disorder, current manic episode with mood-congruent psychotic features (ICD-10-CM F31.2), in which DI constitutes the phenomenological character of the psychotic content. The diagnostic confidence of this primary Bipolar I formulation is appropriately tempered by the patient’s limited retrospective history and the absence of corroborating records. Effective management in this case relied on a multidisciplinary approach focused on mood stabilization and antipsychotic therapy. The concurrent initiation of divalproex and olanzapine, rather than antipsychotic monotherapy, suggests that addressing the underlying mood state was equally vital to stabilizing her somatic delusions. Furthermore, the use of empathetic, nonconfrontational communication validating the patient’s distress without confirming the delusion was essential for building the trust necessary for treatment adherence.

This case contributes to the growing body of literature on secondary DI, particularly in the complex context of comorbid bipolar disorder and stimulant use, a combination that remains underrepresented in published reports. While the patient’s delusional conviction was not fully eradicated, the significant reduction in her distress and the cessation of self-destructive behaviors underscore an important clinical reality: persistent delusional conviction with improved functioning and reduced morbidity is a successful and recognized treatment outcome. Clinicians should note that “delusional infestation” functions in this report as a descriptive term for a cluster of somatic psychotic symptoms, such as formication, fixed parasitic beliefs, and specimen-gathering behavior, rather than as a formal DSM-5-TR diagnosis. The appropriate formal diagnosis in this case is bipolar I disorder with mood-congruent psychotic features, with stimulant use as a precipitating factor. Ultimately, this case emphasizes the necessity of patient-centered communication to foster long-term recovery in a population often alienated by repeated dismissal of their symptoms across multiple medical encounters.
